# The need for unbiased genetic screens to dissect aggression in *Drosophila melanogaster*

**DOI:** 10.3389/fnbeh.2022.901453

**Published:** 2022-08-01

**Authors:** Gary Huang, Herman A. Dierick

**Affiliations:** ^1^Department of Molecular & Human Genetics, Baylor College of Medicine, Houston, TX, United States; ^2^Department of Neuroscience, Baylor College of Medicine, Houston, TX, United States

**Keywords:** aggression, *Drosophila melanogaster*, genetic screen, chemical mutagenesis, P-element insertions

## Abstract

Aggression is an evolutionarily conserved behavior present in most animals and is necessary for survival when competing for limited resources and mating partners. Studies have shown that aggression is modulated both genetically and epigenetically, but details of how the molecular and cellular mechanisms interact to determine aggressive behavior remain to be elucidated. In recent decades, *Drosophila melanogaster* has emerged as a powerful model system to understand the mechanisms that regulate aggression. Surprisingly most of the findings discovered to date have not come from genetic screens despite the fly’s long and successful history of using screens to unravel its biology. Here, we highlight the tools and techniques used to successfully screen for aggression-linked behavioral elements in *Drosophila* and discuss the potential impact future screens have in advancing our knowledge of the underlying genetic and neural circuits governing aggression.

## Introduction

For many decades mutant screens have been a powerful approach to investigate the molecular mechanisms underlying the basic processes of life (Beadle and Tatum, 1941; Tatum and Lederberg, 1947; Hartwell et al., 1970; Hartwell, 1971; Nurse, 1975; Kenyon and Walker, 1980; Schekman et al., 1983; Tsukada and Ohsumi, 1993). Genetic screens only became possible after the discovery that ionizing radiation is mutagenic (Gager and Blakeslee, 1927; Muller, 1927, 1928; Calabrese, 2018), which vastly increased the number of mutants that could be generated and analyzed in a screen. Beadle and Tatum used X-ray mutagenesis in a conceptually simple screen in *Neurospora* to isolate mutants responsible for specific metabolic functions essential for life by switching mutants from complete media to minimal media supplemented with specific metabolites (Beadle and Tatum, 1941). Their screen, in combination with the selection approach isolated strains that were deficient in three essential metabolic enzymes necessary for the synthesis of pyridoxine, thiazole, and para-amino benzoic acid respectively (Beadle and Tatum, 1941). The approach revolutionized the identification of essential genes and has even been credited as the origin of molecular biology (Horowitz et al., 2004; Strauss, 2016). Screens also revealed other basic and universal processes such as gene recombination in *E. coli* (Tatum and Lederberg, 1947), genetic control of cell division (Hartwell et al., 1970; Hartwell, 1971; Nurse, 1975), vesicle secretion (Schekman et al., 1983), and autophagy (Tsukada and Ohsumi, 1993) to name just a few.

The idea turned out to be equally powerful in multicellular organisms. In worms and flies, screens identified the molecular players that control development (Brenner, 1974; Riddle et al., 1981; Jürgens et al., 1984; Nüsslein-Volhard et al., 1984; Wieschaus et al., 1984), cell death (Ellis and Horvitz, 1986), aging (Kenyon et al., 1993), immunity (Lemaitre et al., 1995), and many other biological processes. Many of the genes identified in these screens turned out to be conserved across the animal kingdom (Wieschaus and Nüsslein-Volhard, 2016). One of the pathways uncovered by the screen for embryonic lethal fly mutants is the Toll pathway, which plays a role in dorso-ventral polarity in *Drosophila* embryos (Anderson et al., 1985a, b; Nüsslein-Volhard, 2022). This pathway was later discovered to regulate part of the innate immune responses in flies (Lemaitre, 2004) and eventually led to the discovery of Toll-like receptors (TLRs) as regulators of immunity in mammals (Medzhitov et al., 1997; Takeda and Akira, 2004). Not surprisingly, other genetic model systems, including plants and even vertebrates like zebrafish and mice, have successfully used mutagenesis to uncover basic mechanisms of development and identify unknown gene functions (Patton and Zon, 2001; Page and Grossniklaus, 2002; Kile and Hilton, 2005).

In the 60s, Benzer who had originally used mutant screens to dissect the nature of the gene in phage (Benzer, 1956; Benzer and Champe, 1961) became convinced this approach would allow him to dissect the genetic underpinnings of behavior. Just a few years earlier, Margaret Bastock had shown that a *yellow* pigmentation mutant affected courtship in flies (Bastock, 1956), which was the first demonstration that a single gene can affect behavior. This finding may have inspired Benzer (Cobb, 2007), and his group to embark on a series of screens premised on simple behavioral paradigms in *Drosophila melanogaster* (Greenspan, 1990). This effort again turned out to be very successful and led to the isolation of a series of behavior mutants, one of which was the first courtship mutant isolated from a screen (Gill, 1963). The mutant (*fru^1^*) affected the *fruitless* (*fru*) locus (Ito et al., 1996; Lee and Hall, 2001), which encodes a transcription factor necessary and sufficient for male courtship and mating (Villella and Hall, 2008; Sato and Yamamoto, 2020). In another screen, they isolated the first learning and memory mutant, *dunce^1^* (*dnc^1^*) (Dudai et al., 1976). The *dunce* gene was later cloned and shown to encode cAMP phophodiesterase (Chen et al., 1986), further providing evidence to a growing body of knowledge that cAMP signaling plays an important role in memory formation across species (Alberini, 1999). Perhaps the most famous mutants isolated in those early days were the first circadian mutants (Konopka and Benzer, 1971). In a small screen of less than 2,000 mutants, they identified three alleles that mapped to the *period* locus: one arrhythmic (*per^0^*), one with a short period (*per^S^*), and the last one with a long period (*per^L^*) (Konopka and Benzer, 1971). This simple screen changed the field of circadian biology although it took several more decades before the gene was cloned (Bargiello et al., 1984; Reddy et al., 1984) and the basic aspects of its molecular mechanisms became better understood (Hall, 2005). This process was further aided by additional screens that isolated other components of the pathway (Price, 2005) and by discoveries in mammalian systems, which helped further establish the transcriptional feedback loop that maintains the circadian clock (Honma, 2018).

Despite the potential of mutant screens to elucidate the underlying networks governing many facets of biology, they come with some drawbacks. Arguably the biggest hurdle to overcome in a saturation screen, in particular, is the sheer amount of labor involved. Depending on the number of genes that control the process of interest, performing a screen to saturation can take years (Wieschaus and Nüsslein-Volhard, 2016), and does not guarantee that most or all genes are identified. The workload is based on the genetic effort to make mutant strains (St Johnston, 2002) and on the time it takes to phenotype these mutants. For behavioral screens, this adds an additional layer of difficulty because the organism must be monitored for a sufficient amount of time to ensure that a statistically significantly measurable phenotype can be recorded. Once the phenotype in a specific mutant is confirmed, the causal locus has to be identified. This typically involves a mapping step that can itself be time-consuming (St Johnston, 2002). However, as whole-genome sequencing platforms have become cheaper, mapping can be skipped as long as the screen is deep enough that multiple alleles for each gene can be isolated in so-called complementation groups (Sarin et al., 2008, 2010; Hobert, 2010; Haelterman et al., 2014). When the mutants from the same complementation group are sequenced, only the causal gene will have a deleterious mutation in all the mutants. The assay that is used for phenotyping mutants is also very important for the success of a screen. It is critical that it is sensitive and specific enough to limit both false positives and false negatives. Despite these limitations, the expansive toolkit for genetic and molecular manipulation in fruit flies is one of the major factors enabling screens to be carried out efficiently in this organism even for behavior. This has led to many successful screens that have helped elucidate a range of behavioral phenotypes in flies such as olfaction (Helfand and Carlson, 1989; McKenna et al., 1989), hearing (Eberl et al., 1997), alcohol sensitivity (Singh and Heberlein, 2000), pain (Tracey et al., 2003), sleep (Cirelli et al., 2005; Stavropoulos and Young, 2011), and gravity sensing (Armstrong et al., 2006) to name only a few. Nevertheless, very few screens so far have been done to elucidate complex social behaviors such as aggression.

Here, we highlight the few small-scale unbiased screens (Hoopfer et al., 2015; Davis et al., 2018; Eddison, 2021) that have so far been performed to help figure out the underlying molecular and circuit mechanisms that control aggression in flies to illustrate the power of this approach. We also briefly discuss a recent behavioral pipeline (Chowdhury et al., 2021) that was optimized to perform high throughput screens for aggression in flies to capitalize on the strength of this approach to better understand this elusive phenotype.

## Chemical Mutagenesis Screen for Aggression

The original method to induce random mutations in the genome in most early screens including *Drosophila* was done with ionizing radiation, but most random mutant screens now use chemicals or transposons (see below, Table 1). The most commonly used chemical mutagen in flies is ethyl methanesulfonate (EMS) although other agents can and have also been used (St Johnston, 2002; Greenspan, 2004; Venken and Bellen, 2014). EMS is an alkylating agent that primarily induces random mutations *via* GC > AT transitions although other changes are also possible, including small deletions and insertions (Arrizabalaga and Lehmann, 1999). In the standard protocol male flies are fed a solution of 25 mM EMS in 1% sucrose overnight and crossed to untreated females to produce mutant offspring each unique in their repertoire of induced mutations (Lewis and Bacher, 1968). Lower concentrations have also been used as they lead to a lower number of mutations (Haelterman et al., 2014), which may be beneficial for screens where flies have to behave instead of merely survive. The use of EMS inevitably results in lower mutation rates in small genes creating a bias towards mutating larger genes. Thus, the chances of recovering novel alleles of small genes are very low in a small-scale mutagenesis screen, and often require significantly increasing the number of flies that are mutagenized. Despite this drawback, mutagenesis screens are still useful in identifying novel genes involved in a given process.

**Table 1:** Types of screens for analyzing aggression.

Type of screen	Description	Advantages/Disadvantages
Chemical mutagenesis	Uses chemical mutagens to induce random mutations across the genome. EMS is the most common one in *Drosophila* and induces primarily GC>AT transitions.	Chemical mutagenesis can cause a gain of function as well as loss of function mutations. Many mutations occur per chromosome so lower numbers need to be screened than for transposons. Mapping mutants is hard although this can be circumvented if multiple alleles are isolated for each gene so that sequencing can often identify the causal locus. Genetic background can be controlled by using an isogenized background at the start of the screen.
P-element disruption	Uses transposons to randomly integrate and disrupt gene function. Many different transposons exist with different genome biases.	Transposon insertions almost always lead to loss or partial loss of function phenotypes. Most transposons have insertion bias. Different transposons should be considered to circumvent bias. Typically only one gene at a time is mutated so many more mutants need to be screened to reach saturation. Mapping the mutants is easier than mapping chemically induced mutants. Large collections of transposons exist. The genetic background of different collections may affect behavior.
GAL4/UAS	Collections of P-elements containing GAL4 have been generated to drive the expression of different effectors in particular cell types.	Large GAL4 libraries exist and these can be crossed to many different effectors. Effectors that increase or decrease activity are available and can successfully identify circuits that control aggression. GAL4 lines can also be combined with RNAi lines for which there are also collections. The choice of the driver is important in an RNAi screen because knockdown in the wrong cell type may lead to false negatives. Knockdown is sometimes insufficient to observe a phenotype also causing false negatives.

Genetically the easiest and quickest EMS-induced mutant screen is an F1 screen for viable mutants on the X-chromosome. To make this happen, mutagenized males are crossed to attached-X females (X^X/Y females) and pass on their mutagenized X’s patroclinously to their sons (Bridges, 1914; Morgan, 1922) because the attached-X chromosome passes to the next generation as two X’s, inevitably making a female. To produce fertile males in this cross, the attached-X females also carry a Y-chromosome that they pass on to their sons, making them mutagenized-X/Y and fertile (XO males are sterile). This cross scheme was used in the original circadian mutant screen that identified the three *per* alleles (Konopka and Benzer, 1971). Because aggression cannot be tested on a single male, an extra generation is required by simply crossing each unique F1 mutant male again to X^X/Y females to produce F2 males that all have the same mutant X as their F1 fathers. Rather than testing these F2 males against each other in an assay to measure aggression directly, Davis and colleagues used a secondary phenotype to perform the screen (Davis et al., 2018). They had found that hyper-aggressive males cause wing damage to each other’s wings when they are group-housed. Using this simple proxy phenotype, they screened approximately 1,400 viable fertile mutants and found five that also had an increased aggression phenotype. After performing whole genome sequencing on all the mutants, they found that each had on average about 30 altered coding variations that could be responsible for the mutant phenotype. To rapidly sort through these, they crossed each mutant to a defined set of duplications on the X-chromosome (Venken et al., 2010) that each covered one of the candidate mutations to test whether any of these rescued the mutant phenotype. This strategy led them to find a novel mutation in *Shaker* responsible for one of the mutant’s increased aggression phenotype (Davis et al., 2018). Shaker is the major voltage-gated potassium channel in *Drosophila* but how this mutation affects aggression remains unclear. This screen was relatively fast as the primary screen including mutagenesis and F2 screen took only 3 months while the secondary screen to specifically assess aggression took two more months. However, of the 40 mutant hits in the primary wing damage screen, only five also showed increased aggression. This suggests that there were both false positives and likely false negatives given the low overall number of positives. Clearly, a better primary assay would improve the hit rate on the screen. Below we will discuss a newer assay with high specificity, high sensitivity, and is amenable to high throughput analysis (Chowdhury et al., 2021).

## P-Element Insertion Screens for Aggression

P-elements are transposable elements whose sequence encodes a transposase and terminally located inverted repeats that direct transposition (Engels, 1983). Random insertion of the P-element into a gene can disrupt its function, which has led to its use as a mutagen for screens (Cooley et al., 1988). However, because P-element insertions are biased toward certain regions of the genome (Venken and Bellen, 2014) the most common method of using P-elements in a screen is to screen through P-element insertion libraries. These are collections of flies where each strain contains a P-element insertion in a unique gene. For example, the library generated by the Berkeley *Drosophila* Genome Project comprises 1,045 strains with single P-element insertions disrupting more than 25% of essential genes (Spradling et al., 1999). Many of the insertion sites have been precisely mapped, greatly reducing the amount of time and labor required in traditional mutagenesis screens to pinpoint and characterize the disruptive mutation. Similarly, the Kyoto stock center has a collection of 6,900 PGS lines that were generated by the *Drosophila* Gene Search Project and that can be used for gain-of-function approaches (Toba et al., 1999).

Some P-element insertion libraries are not publicly available but are instead generated and housed by individual labs. Some of these libraries incorporate the *GAL4/UAS* system (Brand and Perrimon, 1993) to allow for a broader range of manipulations than simply disrupting gene function. A recent forward genetic screen used a P[GAL4] insertion library of 1,606 lines with random insertions of *GAL4* throughout the fly genome to screen for mutants with altered behavioral susceptibility to social isolation, including aggression (Eddison, 2021). Forced social isolation in *D. melanogaster* is known to increase aggression levels (Hoffmann and Cacoyianni, 1990; Wang et al., 2008), locomotor activity (Panova et al., 2013), and resistance to ethanol sedation (Eddison et al., 2011), but most of the underlying genes remain unknown. The screen identified a mutant, *sex pistols* (*sxp*) that enhances the effect of social isolation on aggression and resistance to ethanol sedation, and also showed strong male-male courtship. The P-element in this strain is inserted between the first two non-coding exons of *hu-li tai shao* (*hts*, an ortholog of *adducin*) and is close to the start codon of CalpA (encoding a calcium-dependent protease). By capitalizing on the embedded *GAL4* driver in this transposon insertion, the author was able to use RNAi lines against *hts* and *CalpA* to test the causality of the two genes in the phenotypes affected in this mutant to further unravel the phenotype (Eddison, 2021). Knockdown experiments and genomic rescue suggested that the increased aggression phenotype is due to the reduction of *hts* and that increased courtship is due to reduced levels of *CalpA* in *sxp* mutants.

The use of *GAL4* insertion libraries in screens is not limited to just dissecting gene function to identify the genetic interactions that give rise to behaviors, but also to understand the underlying circuits. This is important because understanding the mechanisms that govern behavior not only requires mapping the underlying genetic networks, but also the neural circuitry that drives the behavior. For this reason, a collection of almost 7,000 *GAL4* lines was created using site-specific integration to insert *GAL4* driven by defined genomic DNA fragments from some 1,200 neuronal genes to serve as specific transcriptional enhancers (Jenett et al., 2012). All of these driver lines were analyzed for expression in the adult brain and ventral nerve cord by crossing them to a fluorescent reporter (Jenett et al., 2012). Many of these are likely expressed outside of the nervous system and most of these fragments only show limited resemblance to the expression pattern of the gene from which the fragment is derived, but the collection is nevertheless very useful to investigate circuit connectivity and function. Hoopfer and colleagues used a subset of this collection with the narrowest expression patterns to look for neurons that promote aggression (Hoopfer et al., 2015). They examined aggression in about 2,200 strains that were crossed to *UAS-TrpA1*. Expression of this thermosensitive ion channel increases the neuronal activity in the circuit acutely by raising the temperature of the flies (Hamada et al., 2008). Nineteen of the lines they tested showed a strong increase in aggression at the elevated temperature and three of them also showed increased male-male courtship, a phenotype associated with mutations in the *fruitless* (*fru*) locus, one of the mutants originally isolated in the Benzer lab (Gill, 1963). Two of these showed overlapping expression in a small set of neurons known as P1 neurons, which are part of the *fruitless* circuit in the brain known to play a key role in male-specific behaviors (Villella and Hall, 2008). Depending on the strength of activation of these neurons, the males either engaged in fighting or courtship (Hoopfer et al., 2015). P1 neurons are part of a larger network of so-called pC1 neurons, some of which also express *doublesex*, another sex determination regulator. A subset of these pC1 neurons that are *fru*-negative and *dsx*-positive strongly increase aggression when activated, while *fru*-positive, *dsx*-negative pC1 neurons drive courtship when activated, suggesting this circuit acts as a switch between these normally mutually exclusive behaviors (Koganezawa et al., 2016).

An in-depth analysis of a different line identified in the screen turned out to be important to regulate wing threat (Duistermars et al., 2018), a phenotype also long known to be part of the aggression repertoire in *Drosophila* males (Jacobs, 1960).

## Divider Assay for High Throughput Aggression Analysis

One of the important bottlenecks in genetic screens is the workload to generate the unique mutants and phenotype them. One of the screens (Davis et al., 2018) we highlighted used a simple-to-score wing damage phenotype, which reduced phenotyping workload but also reduced the sensitivity and specificity of the screen. The other two screens (Hoopfer et al., 2015; Eddison, 2021) used existing genetic resources but directly measured aggression by using automated video analysis methods (Dankert et al., 2009; Kabra et al., 2013; Eyjolfsdottir et al., 2014). Despite the automation of behavioral analysis, these screens were still slow because setting up aggression experiments is also labor-intensive. Flies need to be collected, isolated, and loaded in multiple pairs to have enough statistical power to measure a significant phenotypic effect.

**Figure 1:**
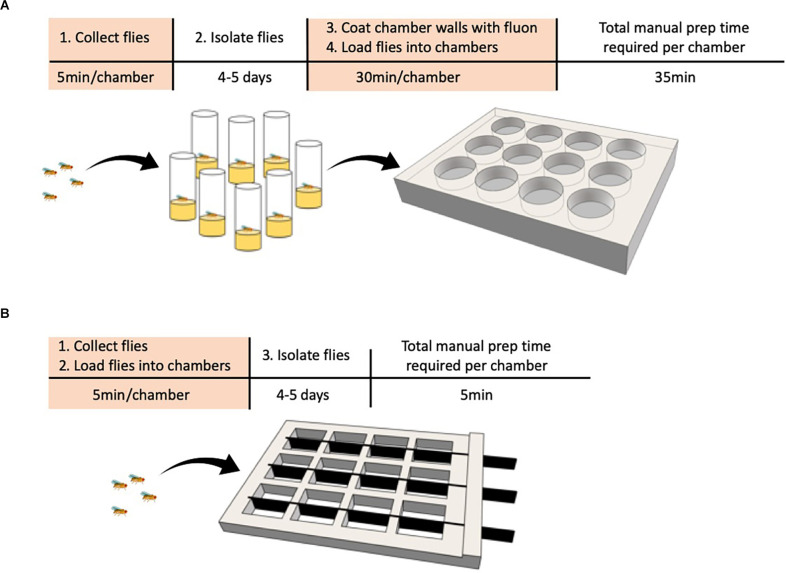
Comparison of behavioral chamber setup to streamline phenotyping in a screen. **(A,B)** Steps requiring manual labor are highlighted in orange. **(A)** Traditional method. The experimenter collects male flies and loads one male fly per vial. The flies are grown in isolation for 4–5 days, then transferred to the behavioral chamber with one pair per circular well before behavior is recorded. **(B)** Divider assay. The experimenter collects male flies and loads two flies per chamber, one on each side of the removable divider. The flies are grown in isolation for 4–5 days, the dividers removed, and behavior is recorded.

A recently developed Divider Assay (Figure 1; Chowdhury et al., 2021) solves the phenotyping bottleneck and should significantly improve the ability to perform high throughput screens. This method uses a cheap 3D printed chamber with 12 square arenas. Each arena can be divided in half by a removable opaque divider allowing each pair of males to be separated on either side of the divider. Flies can now be collected, loaded, and isolated in a single step rather than in separate steps, reducing the time it takes to set up an experiment to just a few minutes. Combined with automated behavioral video analysis that has high specificity and sensitivity, this assay makes phenotyping mutants fast and should be a useful advancement to perform high throughput genetic screens.

## Discussion

Aggression is a complex behavioral phenotype that, despite decades of work, is still not very well understood. In the last two decades, *Drosophila* has emerged as a powerful model to study this behavior. Since its original detailed description (Jacobs, 1960) and repeated rediscovery (Dow and Schilcher, 1975; Hoffmann, 1987a, b, Hoffmann, 1989; Chen et al., 2002), we have learned much about the biology of aggression in this species. Aggression is heritable (Hoffmann, 1988) and selectable (Hoffmann and Cacoyianni, 1989; Dierick and Greenspan, 2006), modulated by biogenic amines and neuropeptides (Certel et al., 2007; Dierick and Greenspan, 2007; Hoyer et al., 2008; Zhou et al., 2008; Alekseyenko et al., 2010, 2014; Andrews et al., 2014; Asahina et al., 2014; Davis et al., 2014; Thomas et al., 2015; Wu et al., 2020), some of which are controlled by conserved transcriptional regulators (Davis et al., 2014; Thomas et al., 2015), influenced by pheromonal cues and receptors (Fernández et al., 2010; Wang and Anderson, 2010; Liu et al., 2011; Wang et al., 2011; Andrews et al., 2014), and controlled by sex determination pathways (Vrontou et al., 2006; Chan and Kravitz, 2007; Asahina et al., 2014; Hoopfer et al., 2015; Koganezawa et al., 2016; Wohl et al., 2020) to name only some of the recent mechanistic discoveries. Many of these findings were discovered by analyzing conserved pathways or by pursuing educated guesses rather than through unbiased screens. In addition, female aggression has also been described in *Drosophila* and found to be both qualitatively and quantitatively different from male aggression (Nilsen et al., 2004; Ueda and Wu, 2009; Schretter et al., 2020).

Despite the strong and long history of *Drosophila* as a genetic model system (Roberts, 2006) with tremendous impact on neuroscience (Bellen et al., 2010) and behavior (Vosshall, 2007), genetic screens have been sparsely used to uncover the mysteries of aggression (Hoopfer et al., 2015; Davis et al., 2018; Eddison, 2021) and so far only in males. This is likely in part due to the workload associated with genetic screens with the added difficulty of the need to carefully measure a complex behavioral phenotype. However, automated video analysis paradigms for aggression have been around for more than a decade (Hoyer et al., 2008; Dankert et al., 2009), and they have been further improved since then (Kabra et al., 2013; Eyjolfsdottir et al., 2014) and yet only three genetic screens have been done. Two of these even used existing strain collections combined with automated video analysis paradigms (Hoopfer et al., 2015; Eddison, 2021), and one of the screens used a simple proxy phenotype as a primary screen (Davis et al., 2018), suggesting phenotyping remains a bottleneck. A recent simplified experimental setup reduces the phenotyping workload further (Chowdhury et al., 2021) and it will be interesting to see if this reduces the roadblocks to future genetic screens.

What should be the focus going forward? History tells us that unbiased genetic screens are the most successful strategy to identify novel mechanisms for any phenotype. Circuit screens will identify the specific circuits that affect behavior and mutant screens will identify the genes that are important for aggression. Many genetic screens look for mutant phenotypes in both directions, but for aggression that is not advisable because increasing aggression in a mutant is much more likely to be specific than decreasing aggression. For example, a mutant that is sick due to a metabolic defect is likely to fight less, but the underlying cause may have nothing to do with regulatory mechanisms of aggression. In addition, the baseline level of aggression of wild-type flies is quite low (although it can be modulated using food and social isolation, and is also dependent on the assay being used) and in order to reliably observe a decrease in the phenotype, it would be necessary to start with a high aggression mutant. This may be a useful strategy but this type of suppressor screen should not be the first priority in screening. As long as we have no good idea how many genes are involved in aggression regulation, a forward genetic screen is the most straightforward strategy to find comprehensive answers. Once a mutant is identified, it has to first be confirmed. A mutant can be confirmed through rescue with a genomic rescue construct or by recreating it in a different strain through genome editing, for example with Crispr/Cas9 (Zirin et al., 2021). An added benefit of using this method is that a *GAL4* can be knocked into the locus and expression of the gene can be evaluated. This can link gene function to circuit function. Structure-function analysis of the gene can further help elucidate the mechanism of action of the gene.

Large-scale screens are daunting because they can take a long time (Wieschaus and Nüsslein-Volhard, 2016). However, they also have significant benefits. First, when enough mutants are screened, multiple alleles will likely be identified. Mutants are placed in complementation groups by crossing them together (St Johnston, 2002). As most mutants are recessive, an outcross to wild-type will make the phenotype disappear. Crossing recessive mutants together will identify the ones that fail to complement. Most of these will be part of the same complementation group and represent different alleles of the same gene. This will significantly simplify finding the causal variant without a time-consuming mapping step through whole-genome sequencing (Sarin et al., 2008, 2010; Hobert, 2010; Haelterman et al., 2014). Sometimes mutants will fail to complement without being alleles of the same gene, known as non-allelic non-complementation or compound haploinsufficiency. These mutants almost certainly are interesting because they likely encode components of a complex and this will give mechanistic insight into pathways that regulate the phenotype. Identifying the expression pattern of such mutants may narrow down the circuit where these genes are important. Most genes are pleiotropic but components of a complex almost certainly work together in the same cells in their effect on the phenotype and finding the overlap in their expression will likely refine the possible circuit involved in the phenotype again linking gene function to circuit function.

While genetic screens are often viewed as fishing expeditions, many have generated discoveries that have transformed our understanding of biology. It is clear that much remains to be discovered in this exciting field and genetic screens will likely play an important role in that endeavor.

## Author Contributions

GH and HD co-wrote the manuscript. All authors contributed to the article and approved the submitted version.

## Conflict of Interest

The authors declare that the research was conducted in the absence of any commercial or financial relationships that could be construed as a potential conflict of interest.

## Publisher’s Note

All claims expressed in this article are solely those of the authors and do not necessarily represent those of their affiliated organizations, or those of the publisher, the editors and the reviewers. Any product that may be evaluated in this article, or claim that may be made by its manufacturer, is not guaranteed or endorsed by the publisher.
